# Functional neural circuits that underlie developmental stuttering

**DOI:** 10.1371/journal.pone.0179255

**Published:** 2017-07-31

**Authors:** Jianping Qiao, Zhishun Wang, Guihu Zhao, Yuankai Huo, Carl L. Herder, Chamonix O. Sikora, Bradley S. Peterson

**Affiliations:** 1 School of Physics and Electronics, Shandong Province Key Laboratory of Medical Physics and Image Processing Technology, Institute of Data Science and Technology, Shandong Normal University, Jinan, China; 2 Department of Psychiatry, Columbia University, New York, NY, United States of America; 3 School of Information Science and Engineering, Central South University, Changsha, China; 4 American Institute for Stuttering, New York, NY, United States of America; 5 Institute for the Developing Mind, Children’s Hospital Los Angeles, CA, United States of America; 6 Department of Psychiatry, Keck School of Medicine at the University of Southern California, Los Angeles, CA, United States of America; Western University, CANADA

## Abstract

The aim of this study was to identify differences in functional and effective brain connectivity between persons who stutter (PWS) and typically developing (TD) fluent speakers, and to assess whether those differences can serve as biomarkers to distinguish PWS from TD controls. We acquired resting-state functional magnetic resonance imaging data in 44 PWS and 50 TD controls. We then used Independent Component Analysis (ICA) together with Hierarchical Partner Matching (HPM) to identify networks of robust, functionally connected brain regions that were highly reproducible across participants, and we assessed whether connectivity differed significantly across diagnostic groups. We then used Granger Causality (GC) to study the causal interactions (effective connectivity) between the regions that ICA and HPM identified. Finally, we used a kernel support vector machine to assess how well these measures of functional connectivity and granger causality discriminate PWS from TD controls. Functional connectivity was stronger in PWS compared with TD controls in the supplementary motor area (SMA) and primary motor cortices, but weaker in inferior frontal cortex (IFG, Broca’s area), caudate, putamen, and thalamus. Additionally, causal influences were significantly weaker in PWS from the IFG to SMA, and from the basal ganglia to IFG through the thalamus, compared to TD controls. ICA and GC indices together yielded an accuracy of 92.7% in classifying PWS from TD controls. Our findings suggest the presence of dysfunctional circuits that support speech planning and timing cues for the initiation and execution of motor sequences in PWS. Our high accuracy of classification further suggests that these aberrant brain features may serve as robust biomarkers for PWS.

## Introduction

Developmental stuttering is a speech disorder in which sounds, syllables, or words are repeated or prolonged, disrupting the normal flow of speech [1–2]. These speech disruptions may be accompanied by behaviors representing effortful motor control, such as rapid eye blinking or lip tremor. Stuttering affects approximately 5 percent of all children at some time in their life, most often between 2 and 5 years of age, when speech and language skills are developing rapidly. Symptoms typically last from several weeks to several years, with adult persistence noted in less than 25% of those affected [3]. Stuttering therefore affects people of all ages, and it often impairs overall quality of life [4].

Prior neuroimaging studies have revealed structural and functional differences in people who stutter (PWS) compared with fluent speakers. The preponderance of evidence currently suggests that the pathogenesis of stuttering involves neural systems involved in the production and control of speech, those within Broca’s region of the inferior frontal gyrus and associated portions of the arcuate fasciculus, as well as their interconnections with cortico-striato-thalamo-cortical (CSTC) loops [5–11]. Voxel-based morphometry studies using anatomical Magnetic Resonance Imaging (MRI) have reported atypical leftward asymmetry [8, 12–13] and increased white matter volumes in the superior temporal gyrus (STG), middle temporal gyrus (MTG), inferior frontal gyrus (IFG), middle frontal gyrus (MFG) and corpus callosum [14–16] of adults who stutter compared with controls. Moreover, children who stutter are reported to have less grey matter volume (GMV) in the IFG, caudate, and putamen [8, 17–19] but more GMV in the right rolandic operculum and STG relative to fluent children [17]. Diffusion Tensor Imaging (DTI) has shown that children who stutter have reduced fractional anisotropy in the region of the left superior longitudinal fasciculus and bilateral corticospinal tracts [18, 20]. Other DTI studies have also shown that adults who stutter have reduced fractional anisotropy (FA) in the left sensorimotor cortex [21–22]. Our recent spectroscopy study in the same participants of the present study reported reduced levels of N-acetyl aspartate (NAA), an index of neural density, in inferior and superior frontal cortices (including Broca’s region) and caudate nucleus, and increased NAA in the posterior cingulate, lateral parietal, and parahippocampal cortices, as well as the hippocampus and amygdala [5].

Functional imaging studies have suggested the presence of a dysfunctional feedforward CSTC network in language learning [23], with these functions extending to linguistic sequencing in a domain-specific manner [24]; because sequencing difficulties during spoken language are the defining hallmark of stuttering, these studies suggest the importance of CSTC circuits in the pathogenesis of developmental stuttering. Positron Emission Tomography [25–27] and functional Magnetic Resonance Imaging (fMRI) studies of stuttering have reported similar patterns of deactivation in the left IFG (Broca’s region), as well as hyperactivation in right frontal operculum, motor areas, basal ganglia, and thalamus in PWS, implicating disturbances in speech planning [28] and speech execution [29] during various speech [26, 30–32] and non-speech tasks [33–34], even for PWS who are non-English speakers [35–36]. In our prior perfusion study of the same participants included in the present study, we detected lower perfusion at rest in PWS compared to fluent controls in Broca's region bilaterally and in the superior frontal gyrus [10]. Perfusion in Broca’s area correlated inversely with the severity of stuttering and extended posteriorly into other portions of the arcuate fasciculus.

Resting state fMRI studies in stuttering children and adults have reported reduced intrinsic functional connectivity in CTSC and auditory-motor cortical loops of the left hemisphere [37–39], suggesting that the neural systems supporting complex and dynamic speech production is dysfunctional in PWS [6, 33]. These studies support the central role that Broca’s region within the IFG, the basal ganglia, and CSTC loops play in the pathogenesis of dysfluent speech.

Despite this progress from neuroimaging in elaborating the brain basis for stuttering, the neural systems that underlie developmental stuttering have not been fully identified. Analyses of functional connectivity using resting-state functional MRI data can be a powerful tool for identifying the intrinsic neural circuits that underlie pathogenic disturbances in neuropsychiatric illnesses. Therefore, we applied independent component analysis (ICA) with hierarchical partner matching (HPM) [40–42] to resting-state fMRI data, aiming to identify altered intrinsic connectivity within cortical-subcortical circuits in a large sample of PWS compared with age- and sex-matched, typically developing (TD) fluent speakers. ICA combined with HPM identifies robust networks of functionally connected brain regions that are reproducible across participants, and it can identify significant connectivity differences across diagnostic groups. We also used Granger Causality (GC) to study the causal interactions (effective connectivity) among the regions that ICA-HPM identified.

One of the main goals of brain imaging and neuroscience is to uncover neural circuits that govern human behaviors and psychiatric disorders, and thereby to identify biomarkers that can robustly predict and identify these behaviors and disorders. Many studies have demonstrated that machine learning techniques are useful for finding potential biomarkers of psychological disorders [43–45]. However, to our knowledge, no studies have been reported that classify PWS and TD controls based on intrinsic brain connectivity. Therefore, we used machine learning to assess how well the ICA measures of functional connectivity and Granger Causality differentiate PWS from TD controls.

Based on prior theoretical models and empirical studies of stuttering, we hypothesized that we would detect brain connectivity differences in PWS in Broca’s region and the associated language loop, and within the cortical-subcortical neural systems that support the components for phonetic encoding, sensory feedback, motor planning, and motor execution of human speech. We further hypothesized that measures of functional and effective connectivity in these systems would discriminate among PWS and TD controls with high accuracy, thereby providing a set of putative biomarkers for stuttering that can be useful for future studies of the pathophysiology and treatment of PWS.

## Materials and methods

### Participants

We recruited 44 participants with developmental stuttering (PWS: 27 males, 17 females, mean age 24.6±11.2 years, range 9–49 years) via advertisements posted on the internet or at local clinics, hospitals, and stuttering support groups. We recruited participants across this wide age range intentionally, to permit assessment of the stability of group differences in imaging measures across the lifespan. All PWS participants, including the adults, were currently symptomatic. A licensed speech-language pathologist diagnosed all stuttering participants before enrollment. The *Kiddie-Schedule for Affective Disorders and Schizophrenia*, *Present and Lifetime Version* was administered to a parent for participants under age 18 [46], whereas the *Structured Clinical Interview for DSM-IV-TR Axis I Disorders* was administered to participants over 18 [47]. Stuttering participants with comorbid disorders (chronic motor tic disorder, N = 2; attention deficit hyperactivity disorder, N = 2; social anxiety disorder, N = 1) were allowed into the study because these are highly prevalent in stuttering speakers; excluding comorbidities would have impaired the generalizability of our findings.

The *Assessment of the Child’s Experience of Stuttering* (ACES) for children who stuttered and the corresponding scale for adults, the *Overall Assessment of the Speaker's Experience of Stuttering* (OASES), were used to evaluate stuttering severity [48], which ranged from mild to severe, with an average severity of 50.8. Approximately 70% of PWS participants endorsed symptoms of moderate severity. We recruited 50 TD controls (31 males, 19 females, mean age 23.1±11.3 years, ranging from 8 to 49 years) randomly from a telemarketing list of 10,000 names in the local community, excluding those with lifetime Axis I or language disorder, group-matching to the PWS group on age and sex.

The Institutional Review Board of the New York State Psychiatric Institute approved the study procedures. We obtained written informed consent from all participants and parental consent for participants under age 18.

### Image acquisition

Participants were positioned in the scanner and head coil using canthomeatal landmarks. We instructed the participants to keep their eyes closed and not to think about anything in particular for the duration of the scan. Images were acquired using a 3 Telsa GE Signa EXCITE scanner (General Electric, Milwaukee, USA) equipped with an 8-channel head coil. Resting-state fMRI data were acquired using a gradient-echo echo-planar imaging (EPI) sequence (matrix 64×64, field of view 24cm×24cm, repetition time 2200msec, echo time 30msec, flip angle 90°, slice thickness 3.5mm without gap). Thirty-four interleaved slices were acquired along the AC-PC plane to provide whole brain coverage, with 146 images acquired per participant, for a scan duration of 5 minutes 21 seconds.

### Data analysis

#### Preprocessing

Functional MRI data were preprocessed using SPM8 (Welcome Department of Imaging Neuroscience, London, United Kingdom; see http://www.fil.ion.ucl.ac.uk/spm/) that was run under MATLAB. The first six volumes of scans were excluded from analysis to allow for signal stabilization following onset transients. The remaining functional scans (140 volumes) were slice timing-corrected, realigned to the first scan using rigid-body transformations to correct for head movements. Realigned functional volumes were motion-adjusted. The outlier volumes were identified based on the first-order derivative of the motion parameters between successive EPI images and global mean BOLD signal. We used linear interpolation to replace the identified outlier volumes with the closest non-outlier volumes in ArtRepair toolbox version 4 (http://spnl.stanford.edu/tools/ArtRepair) [49]. Finally, functional images were normalized to the Montreal Neurological Institute (MNI) coordinate system [50] and spatially smoothed using an isotropic 8 mm full-width at half-maximum Gaussian kernel.

#### Independent component analysis with hierarchical partner matching

We elected to employ single-subject ICA in this study because, compared with group-based ICA [51], it better preserves the specificity and independence of independent components (ICs) identified within individual participants. Furthermore, ICA combined with HPM is able to identify networks of functionally connected brain regions that are reproducible across participants and that are highly robust with respect to the number of ICs generated for each person [40–41], a considerable advantage that group-based ICA by definition cannot provide. The combination of ICA with HPM, and the validation of the ICs they identify, are provided in detail elsewhere [40–41], but we briefly summarize them here.

Determining rationally and accurately the number of components that should be extracted in any given dataset is notoriously difficult. We therefore combined individual-level ICA with HPM to ensure that we identified ICs that were robust with respect to the number of ICs generated in each participant. HPM first identifies ICs that are robust and reproducible across participants for a given number of extracted ICs, and then it identifies those ICs that are independent of the number of ICs extracted in each participant. Thus in the first HPM step we combined Minimum Description Length and Akaike’s Information Criterion to establish the lower and upper bounds (determined to be 20 and 130, respectively) for the number of ICs likely present in each participant. Next, starting from the lower bound of 20, we increased by increments of 10 the number of IC’s extracted in each person (i.e., 20, 30, 40, 50, 60, 70, 80, 90, 100, 110, 120, and 130 IC’s per set), yielding 12 sets of ICs for each participant. We used Principal Component Analysis (PCA) to do the data reduction prior to the ICA. We reduced the data X with a dimension of T×M to a dimension of N×M, where T represented the number of time points and N represented the number of independent components to be generated (N<<T), and M is the number of the voxels. Spatial ICA was then performed on the reduced N time-point dataset for each subject using the ICA algorithm ‘FastICA’ [52]. In this way, we applied the ICA to individual-level fMRI time series to generate the requisite number of spatial IC’s within each of these 12 sets of IC’s for each participant.

In the first step of HPM we used bidirectional mapping within each of these corresponding 12 sets of ICs to identify components that were reproducible across participants for a given number of components generated for each person (i.e., in the set containing 20 components, then 30 components, and so on). We operationally defined ‘*bidirectional matching’* as follows: Given a component *i* from set A, we calculated Tanimoto Distance which was used as indices of spatial similarity between IC *i* and each IC in set B. Among all the ICs of set B we found an IC, say IC *j*, which had the largest similarity index to IC *i*. Then we calculated all the similarity indices between component *j* in set B and each of the ICs in set A. We selected an IC, say IC *k* in set A that had the largest similarity index to component *j*. If *k* = *i*, then the spatial matching of component *k* in set A and component *j* in set B was bidirectional, and the two components were considered to be bidirectional matched or partner-matched [40]. The ICs that were identified as being significantly similar to one another in their spatial configuration thereby formed a *cluster* within each of the 12 IC sets. We then applied a one-sample *t* test to the *z*-score maps of ICs within each cluster to obtain a map of voxels in which functional connectivity differed significantly from zero within that cluster, which we designated as a ‘*cluster map*’ of reproducible ICs within each of the 12 IC sets.

We next wanted to identify those ICs that were reproducible across participants not only within a single set (identified in the first HPM step), but that were also present regardless of the number of ICs extracted from the original dataset. Therefore in the second HPM step we applied bidirectional mapping a second time, but this time across the 12 sets of ICs, to identify the most similar and reproducible clusters across individuals and across these 12 sets of ICs. We used Cronbach’s Alpha reliability index in each application of bidirectional mapping to identify clusters with the greatest reproducibility across participants both within a given IC set (HPM step one) and across the 12 sets of ICs (HPM step two). Finally, we generated *z*-score maps of the 12 clusters of ICs to represent the strength of connectivity within ICs that are reproducible across participants and that are as independent as possible of the number of ICs generated for each participant.

#### Group-level statistical analyses

The *z*-score maps of the 12 clusters of ICs were entered into a second-level factorial analysis to detect the random effects of group difference in functional connectivity between PWS and TD controls while covarying for age and sex. We used a combination of uncorrected *p*-value of 0.01 and cluster extent threshold of 20 voxels (determined by Monte Carlo simulation) to correct for multiple comparisons across the imaging volume. In addition, we calculated the Pearson’s correlation for these *z*-scores and overall symptom severity (combining across the child and adult scales, then standardized using a *z*-transform), the most representative index of speech difficulties in various social settings. We also assessed the significance of the main effects of age in our model and, in a separate analysis, the significance of an age-by-diagnosis interaction.

#### Assessing the effective connectivity between differing independent components

We used Granger Causality to assess the causal influences between the ICs that we identified using HPM. We extracted the time courses of these components to calculate Granger Causality Indices (GCIs) between ICs [41]. We then used 2-sample *t*-tests to detect group difference in GCIs between PWS and TD controls.

#### Machine learning

We used this pattern classification technique to determine whether the abnormalities we identified in neural connectivity can serve as a functional biomarker for stuttering. The *z-*score maps of ICs and GCIs that differed significantly across diagnostic groups were selected as feature vectors. A kernel support vector machine (SVM) with leave-one-out cross-validation was used to distinguish PWS from TD controls [53]. A Gaussian kernel with width of 0.5 was used in the SVM modeling.

## Results

### Reproducible independent components

**Fig 1:**
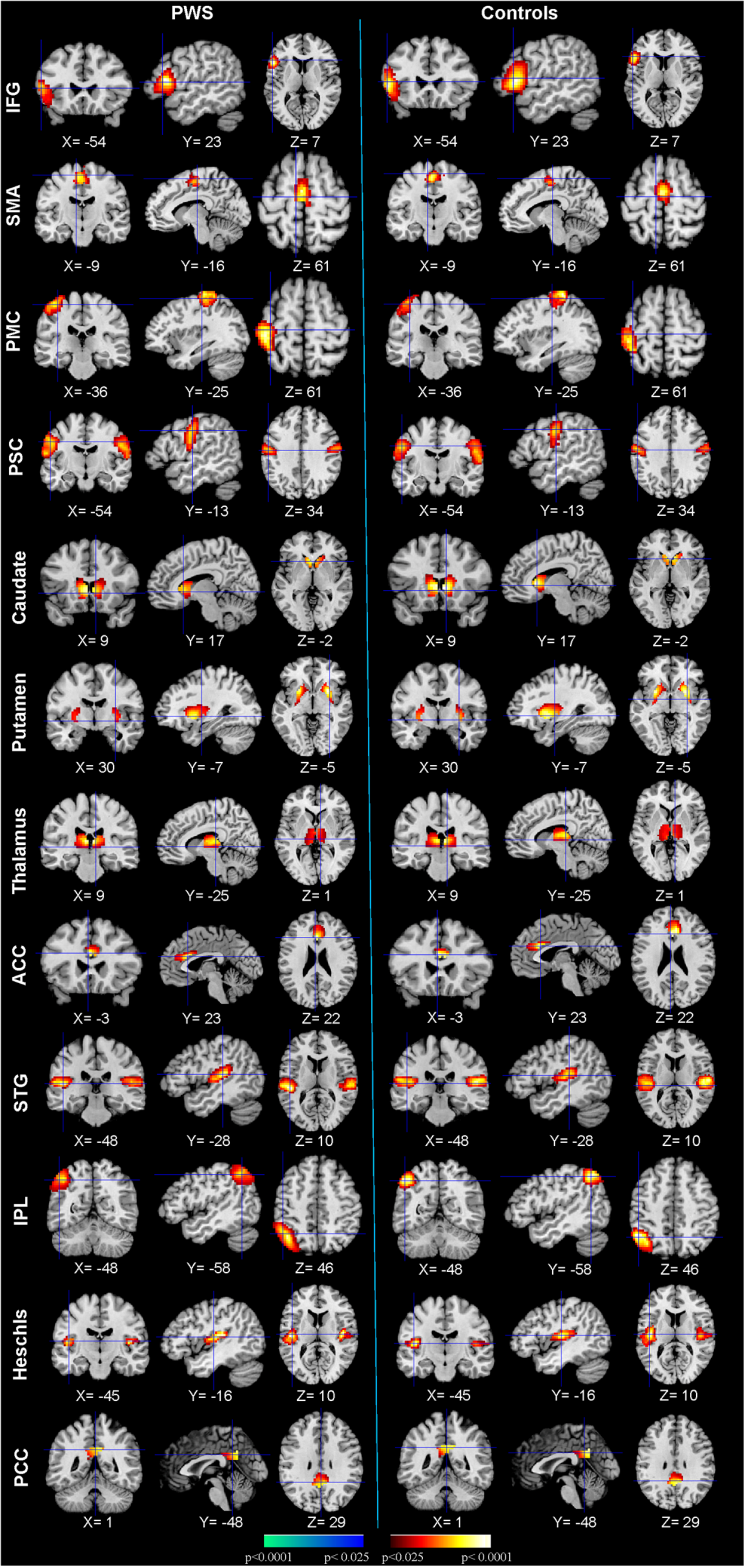
Activity in each of the 12 clusters of reproducible independent components. The first three columns display the random-effect group activity maps detected from the 44 PWS. The first column is a coronal view, the second is a sagittal view, and the third is an axial view. The last three columns displays the group activity maps detected from the 50 TD controls. Each row displays one group activity map generated by applying a one-sample t-test to 1 of the 12 clusters of independent components. Any two group activity maps within the same row across the first three and second three columns are significantly similar to one another in their spatial configurations. PMC = primary motor cortex; SMA = supplementary motor cortex; IFG = inferior frontal gyrus; PSC = primary somatosensory cortex; ACC = anterior cingulate cortex; STG = superior temporal gyrus; IPL = inferior parietal lobule; PCC = posterior cingulate cortex.

By design, spatial ICA generated 12 different sets of ICs for each participant. We applied HPM to these 12 sets of components and identified 10 clusters of ICs that were significantly reproducible in their spatial patterns across stuttering and control participants and across the differing number of ICs extracted from each participant. We performed a one-sample t-test on each of the 10 clusters to generate IC maps that represented statistically significant functional connectivity in this dataset (Fig 1). These maps yielded 12 ICs located in primary motor cortex (PMC), supplementary motor cortex (SMA), inferior frontal gyrus (IFG), primary somatosensory cortex (PSC), caudate, putamen, thalamus, anterior cingulate cortex (ACC), superior temporal gyrus (STG), inferior parietal lobule (IPL), Heschl’s gyrus, and posterior cingulate cortex (PCC).

### Comparing functional connectivity between PWS and TD control participants

**Fig 2:**
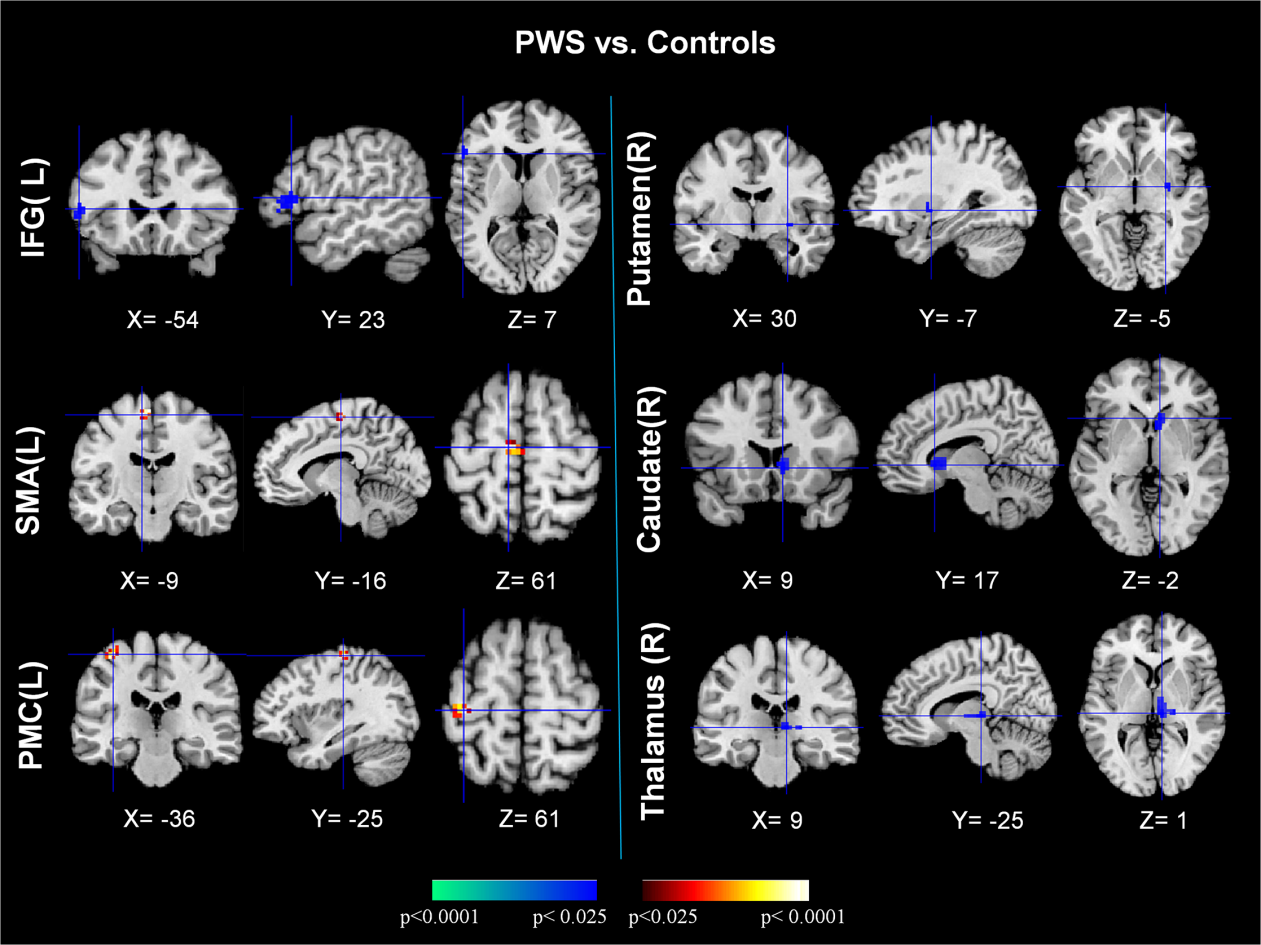
Comparisons of neural connectivity between PWS and normal controls (PWS vs. controls). The two sets of three columns display t-contrast maps comparing the group activity maps from the PWS and TD control participants. The images show that functional connectivity was greater in PWS compared with normal control participants in the SMA and PMC (shown in red), but their functional connectivity was weaker in the IFG, caudate, putamen, and thalamus (shown in blue). PMC = primary motor cortex; SMA = supplementary motor cortex; IFG = inferior frontal gyrus.

**Fig 3:**
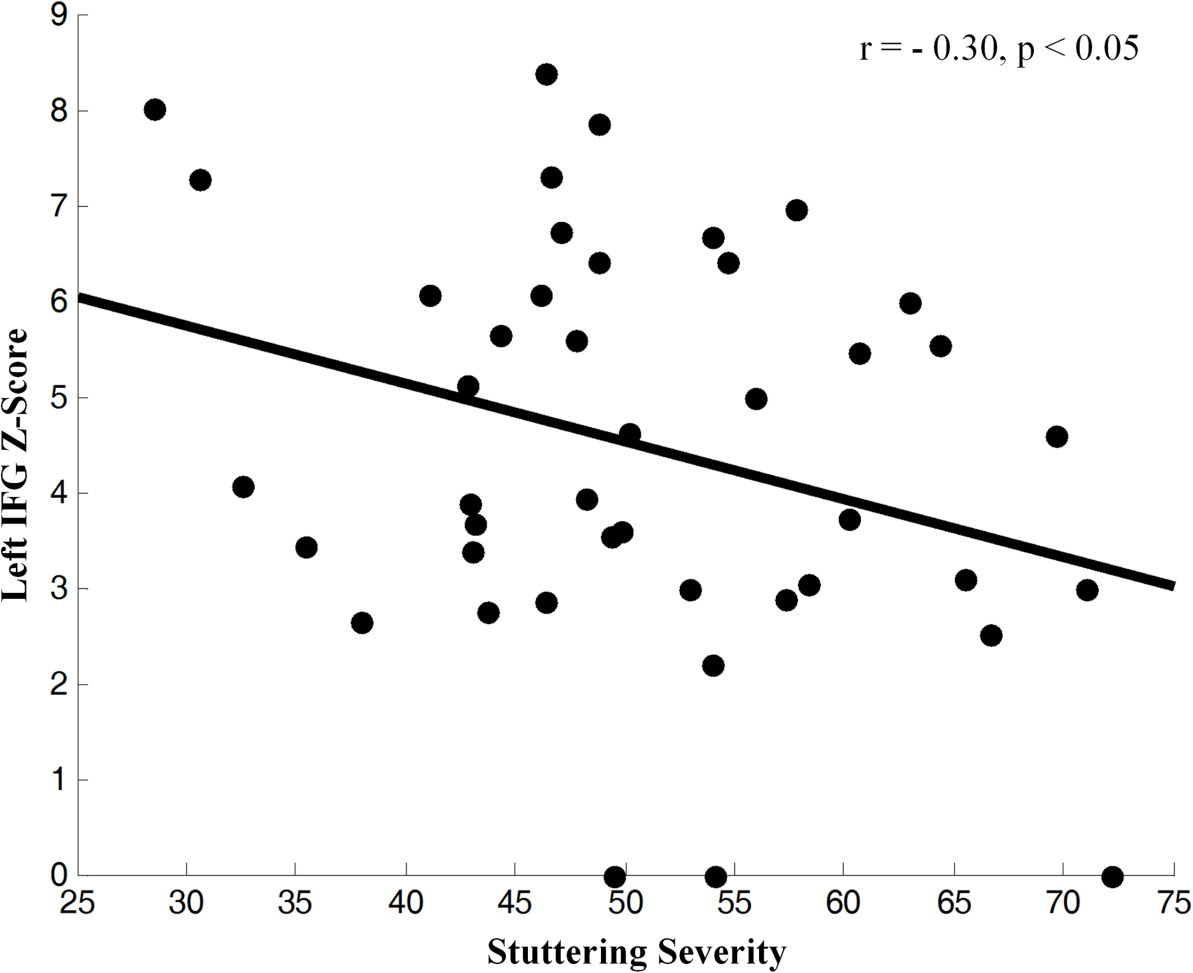
Correlation of z-score for Blood-Oxygen-Level-Dependent (BOLD) activity, an fMRI index of neural activity, in the independent component from the left Inferior Frontal Gyrus (IFG, Broca’s area) with the severity of stuttering symptoms in PWS (z-transformed total score in the ACES and OASES self-report measures). BOLD correlated inversely with symptom severity, indicating that greater neural activity accompanied less severe symptoms across PWS participants.

The reproducible IC’s of PWS and TD controls were compared in a second-level, random-effects analysis while covarying for age and sex. Connectivity in PWS was significantly weaker than in TD controls in the left inferior frontal gyrus (IFG), right putamen, right caudate, and right thalamus. Functional connectivity was significantly stronger in PWS in the left supplementary motor area (SMA) and primary motor cortex (PMC) (Fig 2). The detailed findings are summarized in Table 1. Connectivity in the left IFG correlated inversely with the severity of PWS (r = -0.30, p<0.05) (Fig 3). We detected no significant main effect of age on our measures of functional connectivity, nor did we detect any significant age-by-diagnosis interaction, suggesting that group differences in connectivity were stable throughout development.

**Table 1:** Regional locations and significant comparisons of the independent component maps between PWS and normal control participants.

Brain Areas	Location		Peak location			T	Classification
	Side	BA	x	y	z	statistic	contribution
**PWS vs. Controls (positive)**							
Primary motor cortex (PMC)	L	4	-36	-25	61	+3.06	0.3177
Supplementary motor area (SMA)	L	6	-9	-16	61	+3.81	0.8095
**PWS vs. Controls (negative)**							
Inferior frontal gyrus (IFG)	L	44/45	-54	23	7	-3.09	0.3047
Putamen	R	NA	30	-7	-5	-2.96	0.2550
Caudate	R	NA	9	17	-2	-3.14	0.2213
Thalamus	R	NA	9	-25	1	-3.08	0.1725

NA = Not applicable

All coordinates are in the MNI (Montreal Neurological Institute) ICBM152 space.

#### Granger causality analyses

**Fig 4:**
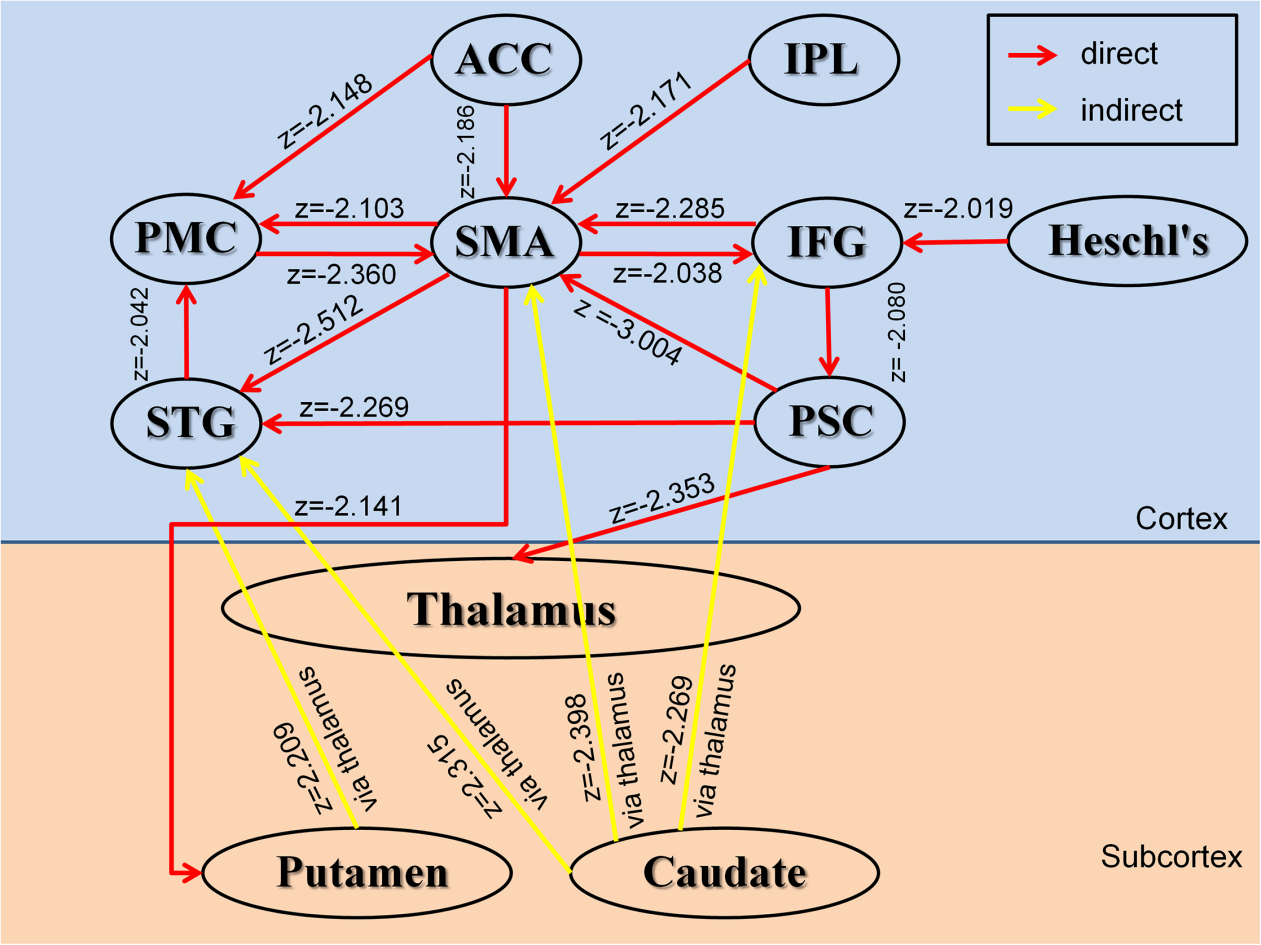
Diagram showing the significant interregional causal connections as estimated by the Granger Causality Index (GCI) and the comparison of GCIs between the PWS and normal control participants (as shown by the corresponding z-score). Red lines represent causal influences from region X to region Y. Yellow lines represent up causal influence from region X to region Y via the thalamus. The arrowhead shows the direction of each causal influence. The z-score indicates the group difference in GCIs between PWS and Controls. Only significant connections are shown.

We computed two types of Granger Causality indices. One was the influence of region A on B, and the other was the influence of region A on B through the thalamus. Causal influences were generally weaker in PWS than in TD controls in connections from the IFG (Broca’s area) to SMA, ACC to SMA, SMA to PMC, SMA to putamen, and caudate to SMA via the thalamus (Fig 4, Table 2).

**Table 2:** Comparisons of statistically significant granger causality indices of the interregional connections of the reproducible independent components.

	PWS	Normal Controls	PWS vs. Controls	Classification contribution
IFG → SMA	0.028 (-0.032–0.089), p = 7.62e-09	0.045(-0.051–0.142), p = 7.56e-10	z = -2.285, p = 2.23E-2	0.1620
PMC → SMA	0.028(-0.053–0.109), p = 7.62e-09	0.066 (-0.033–0.164), p = 7.56e-10	z = -2.360, p = 1.83E-2	0.0909
ACC → SMA	0.058(-0.024–0.140), p = 7.62e-09	0.082(-0.008–0.173), p = 7.56e-10	z = -2.186, p = 2.88E-2	0.0523
PSC → SMA	0.019(-0.020–0.058), p = 7.62e-09	0.041(-0.039–0.121), p = 7.56e-10	z = -3.004, p = 2.70E-3	0.1050
IPL → SMA	0.028(-0.030–0.087), p = 7.62e-09	0.054(-0.048–0.156), p = 7.56e-10	z = -2.171, p = 2.99E-2	0.0049
ACC → PMC	0.115(0.006–0.224), p = 1.12e-08	0.156(0.053–0.259), p = 7.56e-10	z = -2.148, p = 3.17E-2	0.1094
STG → PMC	0.065(-0.047–0.177), p = 7.62e-09	0.122(-0.017–0.261), p = 7.56e-10	z = -2.042, p = 4.11E-2	0.0071
SMA→ PMC	0.111(0.015–0.206), p = 7.62e-09	0.146 (0.022–0.271), p = 7.56e-10	z = -2.103, p = 3.55E-2	0.1258
Heschl's → IFG	0.093(-0.019–0.204), p = 1.12e-08	0.121(0.032–0.210), p = 7.56e-10	z = -2.019, p = 4.34E-2	0.0008
SMA → IFG	0.094(0.004–0.184), p = 1.12e-08	0.133(-0.017–0.282), p = 1.11e-09	z = -2.038, p = 4.15E-2	0.0681
IFG → PSC	0.084(-0.047–0.214), p = 7.62e-09	0.130(0.014–0.245), p = 7.56e-10	z = -2.080, p = 3.75E-2	0.0105
SMA → STG	0.044(-0.044–0.131), p = 1.12e-08	0.116(-0.001–0.232), p = 7.56e-10	z = -2.512, p = 1.20E-2	0.0057
PSC → STG	0.036(-0.021–0.093), p = 7.62e-09	0.077(-0.032–0.185), p = 7.56e-10	z = -2.269, p = 2.32E-2	0.0076
PSC → Thalamus	0.056(-0.046–0.158), p = 7.62e-09	0.102 (-0.009–0.214), p = 7.56e-10	z = -2.353, p = 1.86E-2	0.0128
SMA → Putamen	0.046(-0.016–0.108), p = 7.62e-09	0.077(-0.045–0.199), p = 7.56e-10	z = -2.141, p = 3.23E-2	0.0957
Caudate → SMA via Thalamus	0.022(-0.022–0.065), p = 7.62e-09	0.053(-0.012–0.117), p = 7.56e-10	z = -2.398, p = 1.65E-2	0.0885
Caudate → IFG via Thalamus	0.021(-0.028–0.070), p = 7.62e-09	0.042(-0.026–0.111), p = 7.56e-10	z = -2.269, p = 2.32E-2	0.0901
Caudate → STG via Thalamus	0.080(-0.001–0.161), p = 7.62e-09	0.043 (-0.039–0.125), p = 7.56e-10	z = 2.315, p = 2.06E-2	0.0117
Putamen → STG via Thalamus	0.040(-0.042–0.121), p = 7.62e-09	0.022(-0.025–0.070), p = 7.56e-10	z = 2.209, p = 2.72E-2	0.0109

The data in cells of the second and third columns represent the median and interquartile range of the Granger causality indices. The p-values (uncorrected) indicate how significantly different the median was from zero (assessed using the Wilcoxon signed rank test). We used a two-sided Wilcoxon rank sum test to compare the Granger causality indices of stutterers and controls, yielding the significance test statistics in the fourth column (p<0.05, uncorrected). Thalamus X→Y represents connectivity between X and Y via the thalamus.

### Machine learning classification

We applied kernel SVM to the measures of functional connectivity and GCIs that differed significantly across diagnostic groups, including functional connectivity measures of the IFG, caudate, putamen, thalamus, SMA, and PMC (Table 1) and the GCIs shown in Table 2. Average classification accuracy was 90.3% under a 5-fold cross-validation and 92.7% under a leave-one-out cross-validation. To assess further the respective contributions of these independent components to the classification, we also performed classification using a maximum uncertainty linear discriminant analysis (MLDA) classifier [54] with leave-one-out cross validation, achieving a classification accuracy of 89.9%, with a sensitivity of 82.8% and a specificity of 91.8%. The contribution of each feature to the classification (Tables 1 and 2) was determined using the coefficients of the discrimination hyperplane that measured the weights of the feature in question to the classification. The SMA, PMC, IFG, and putamen made the largest contributions to the classification and can be regarded as the optimal feature subset. Using features of IC activity in the key portions of circuits comprising motor portions of cortical language systems (IFG, PMC, SMA), as well as all available subcortical components of the CTSC loops (caudate, putamen, and thalamus) and GCIs, an average of 1,000 leave-one-out cross validation trials yielded a classification accuracy of 86.8%.

## Discussion

We compared intrinsic neural connectivity between PWS and TD control participants. The ICs that we identified included the inferior frontal gyrus (IFG, Broca’s area), supplementary motor cortex (SMA), primary motor cortex (PMC), anterior cingulate cortex (ACC), posterior cingulate cortex (PCC), primary somatosensory cortex (PSC), inferior parietal lobule (IPL), superior temporal gyrus (STG), and Heschl's gyrus, as well as in the caudate, putamen, and thalamus. Functional connectivity was significantly weaker in PWS in the left IFG and basal ganglia but stronger in the left SMA and PMC. Granger causality influences were generally weaker in PWS than in TD controls in connections from the IFG (Broca’s area) to SMA, ACC to SMA, SMA to PMC, SMA to putamen, and caudate to SMA via the thalamus. As we will elaborate, these findings taken together suggest the presence of dysfunction in the language circuits that support speech planning and the timing, initiation, and execution of motor sequences in PWS [37, 55–59]. The high accuracy of our machine learning classifier applied to these measures of connectivity and causality differences suggests that these brain features may serve as robust biomarkers for PWS.

The IFG (Broca’s area) is a key component of the language network that participates in speech planning, semantic, syntactic, and phonological processing [60–62]. The weaker intrinsic functional connectivity within the IFG of PWS may contribute to their speech disfluency. The significant inverse correlation of connectivity measures with the severity of stuttering symptoms suggests that the more abnormal the connectivity is within the IFG, the more severe were the symptoms in PWS, further supporting a role for reduced connectivity of the IFG in the pathogenesis of stuttering. The reduced causal influence from the IFG to SMA furthermore suggests that at least one locus of network dysfunction in PWS resides in the interface of speech planning processes with motor execution. These findings are consistent with previous anatomical studies reporting reduced gray matter volumes and reduced fractional anisotropy in the left or bilateral IFG [17–19], as well as with previous fMRI studies reporting lower intrinsic functional connectivity in the left IFG [39] and deficient left hemisphere connectivity between inferior frontal and premotor cortices under auditory and visual stimulation [63]. Our finding of reduced intrinsic functional connectivity of the IFG is also consistent with our prior findings of reduced perfusion and reduced spectroscopic indices of neural density in Broca’s region in the same sample of participants as included in the present study [5, 10].

Functional connectivity was also lower in the PWS group compared with TD controls in the caudate, putamen, and thalamus but increased in the left SMA and PMC. The basal ganglia support the learning [64], planning, and internal timing of automatized behaviors [9]. Basal ganglia dysfunction can therefore disrupt the initiation of speech processing [6]. PWS have been reported previously to have reduced volumes [8] and activity [65] in the caudate nucleus. Our Granger Causality analyses further suggest that the causal influence from the caudate to SMA via the thalamus was weaker in PWS than in TD controls. The caudate is generally considered to represent the cognitive control portion of CSTC circuits [66]. Therefore, reduced connectivity of the caudate may represent the presence of deficient control over speech motor programs. Meanwhile, the increased functional connectivity within the SMA and PMC may represent greater effort to control motor behavior in PWS as a compensatory neural response to the deficient input from the caudate.

We also identified ICs in the primary somatosensory cortex (PSC), superior temporal gyrus (STG), inferior parietal lobule (IPL), Heschl’s gyrus, anterior cingulate cortex (ACC) and posterior cingulate cortex (PCC) (Fig 1). Although we did not detect statistically significant differences between the PWS and TD groups in functional connectivity within these ICs, Granger Causality analyses did demonstrate significantly reduced effective connectivity from the PSC, STG, IPL, and ACC to motor areas (Table 2). Previous research has demonstrated that the ACC plays an important role in cognitive control [67]. Less effective connections from the ACC to SMA and PMC may impair intrinsic control of motor execution. Our findings of aberrant connectivity in motor, auditory, sensory, and language areas in PWS are thus in general agreement with the two-loop timing model of speech output [56], which posits that stuttering may be the product of dysfunction within two neural networks: an outer linguistic cortical loop that supports the control of speech-language functions and auditory self-monitoring, and an inner phonatory loop of cortical and subcortical motor regions that controls the output of speech [6, 37]. Disturbances in functional and effective connectivity within the outer or inner loop may disrupt the coordinated activity of these two circuits to produce a momentary instability in coordinated neural activity within these systems that manifests as stuttering behavior [56].

The connectivity and causal indices that differed significantly between groups yielded impressive group discrimination using machine learning, with classification accuracies of 90.3% under a 5-fold cross-validation and 92.7% under a leave-one-out cross-validation. An MLDA classifier suggested that the IFG, SMA, PMC, and putamen together compose an optimal feature subset that made the largest contributions to classification accuracy. The high classification accuracy of these connectivity and causality measures across our diverse sample of participants, including both children and adults across a wide age range, suggests that the measures capture trait-like, rather than state-like, disturbances in PWS, and that the measures are promising functional biomarkers for future studies of the pathogenesis and treatment of stuttering.

Several limitations of this study should be noted. First, the age range of the participants was wide. This was by design, however, so as to assess whether group differences in our connectivity measures varied with age or were stable across the lifespan. The absence of a significant age-by-diagnosis interaction suggests that the findings were stable throughout development, as do our overall accuracy measures for machine learning-based group discrimination. Second, our measures of stuttering severity, the ACES and OASES, are self-reports that provide subjective measures of stuttering severity. Objective measures of speech fluency should be included in future studies. Third, our findings conceivably could have been influenced by the presence of comorbid illnesses in the 5 stuttering participants who had them. Findings were unchanged, however, when excluding those participants from statistical analyses, making us confident that our findings represent stuttering effects rather than comorbidities. Finally, stuttering persons could have had atypical neural responses to MRI scanner noise, which could have yielded atypical measures of brain connectivity. It will be important to replicate our findings using other techniques, such as EEG.

## Supporting information

S1 File Image data. (RAR)
